# Exploring school environmental psychology in children and adolescents: The influence of environmental and psychosocial factors on sustainable behavior in Indonesia

**DOI:** 10.1016/j.heliyon.2024.e37881

**Published:** 2024-09-12

**Authors:** Tia Rahmania

**Affiliations:** Department of Psychology, University of Paramadina, Jakarta, Indonesia

**Keywords:** Environment, Psychosocial factors, Sustainable behavior, Indonesia

## Abstract

This study investigates the relationship between environmental and psychosocial factors and students' sustainable behavior in schools. Through a mixed-methods approach, including surveys, interviews, observations, and document analysis, it explores various dimensions such as the physical environment, policy and governance, social and cultural context, economic factors, technological advancements, stakeholder engagement, knowledge and awareness, attitudes and values, perceived behavioral control, social norms and influence, motivation and incentives, and social identity and connectedness. Findings emphasize the crucial role of the school environment in shaping sustainable behavior and advocate for targeted interventions and policies. Implications include the development of sustainable school environments and interventions fostering positive attitudes and behaviors towards sustainability. This research provides practical insights for educators, policymakers, and stakeholders involved in promoting sustainability in schools.

The school environment plays a significant role in shaping the development of children and adolescents [1,2]. It not only provides formal education but also serves as a social setting where young individuals spend a substantial amount of their time [[3], [4], [5]]. Creating a sustainable school environment is crucial for instilling sustainable values, attitudes, and behaviors in children and adolescents, fostering their understanding of environmental responsibility ([6,7]; H. [8]). In recent years, there has been a growing recognition of the importance of sustainability on a global scale [9]. The urgent need to protect the environment and achieve sustainable development has led to efforts to integrate sustainability into various aspects of society, including schools [[10], [11], [12]]. Schools have the potential to be powerful agents of change, equipping future generations with the knowledge and skills needed to address environmental challenges [13].

Despite the increasing focus on sustainability in schools, there remains a research gap concerning the psychological factors that influence sustainable behavior among children and adolescents. Sustainable psychology explores how individual perceptions, motivations, attitudes, and values shape sustainable behavior [14,15]. Understanding these psychological factors within the school environment is crucial for developing effective interventions that can promote sustainable behavior among students [16]. Furthermore, the physical and social environment within schools also plays a significant role in influencing sustainable behavior. Creating an environmentally friendly infrastructure, implementing sustainable practices, and integrating sustainability across the curriculum can have a profound impact on students' attitudes and behaviors towards the environment. By providing a supportive and sustainable school environment, we can foster a sense of responsibility and stewardship in children and adolescents.

In other hand, Indonesia is chosen as the focus of this research for several significant reasons. Firstly, as a developing country with a large population and rapid economic growth, Indonesia provides a relevant insight into how environmental and psychosocial factors influence sustainable behavior amidst complex socio-economic dynamics. For instance, rapid population growth, urbanization, and significant industrial expansion in Indonesia have posed serious challenges related to environmental degradation, climate change, and sustainable resource management. Secondly, Indonesia boasts rich environmental and cultural diversity, ranging from tropical rainforests to coral reefs, from bustling metropolises to remote rural areas. This diversity creates opportunities for diverse and representative case studies, allowing for an understanding of how environmental and psychosocial factors interact in different contexts. Lastly, Indonesia has taken significant steps to strengthen environmental education in schools and implement sustainability policies across various sectors. For example, the implementation of the 2013 Curriculum in Indonesia has promoted environmental education as a crucial component of primary and secondary education curricula. This offers an opportunity to study the impact of these efforts in stimulating sustainable behavior among students, as well as the effectiveness of policies and programs implemented to promote sustainability at local, national, and global levels.

Meanwhile, the Environmental Quality Index (EQI) in Indonesia over the past nine years shows a fluctuating trend with both increases and decreases in values. According to katadata (2024), in 2015, the EQI stood at 68.23, indicating a relatively moderate environmental quality level. Subsequently, in 2016, there was a slight decrease to 65.73, suggesting a deterioration in environmental conditions. However, the following year, in 2017, there was a slight improvement, with the EQI rising to 66.46. The trend continued with a decrease in 2018 to 65.14, followed by a notable increase in 2019 to 66.55, indicating some variability in environmental quality during this period. However, from 2020 onwards, there is a clear upward trend in the EQI, signifying consistent improvements in environmental quality over the past three years. In 2020, the EQI rose to 70.27, followed by further increases in 2021 (71.45) and 2022 (72.42). The trend culminated in 2023 with an EQI of 72.54, representing the highest level of environmental quality observed during this period. Overall, the data suggest a positive trajectory towards enhanced environmental quality, particularly in the recent years leading up to 2023 (see Fig. 1) (see Fig. 2).

**Fig. 1:**
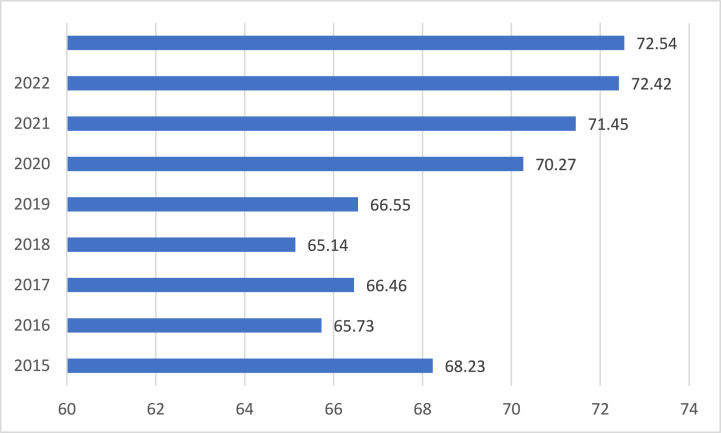
Environmental Quality Index in Indonesia (source: [17]).

**Fig. 2:**
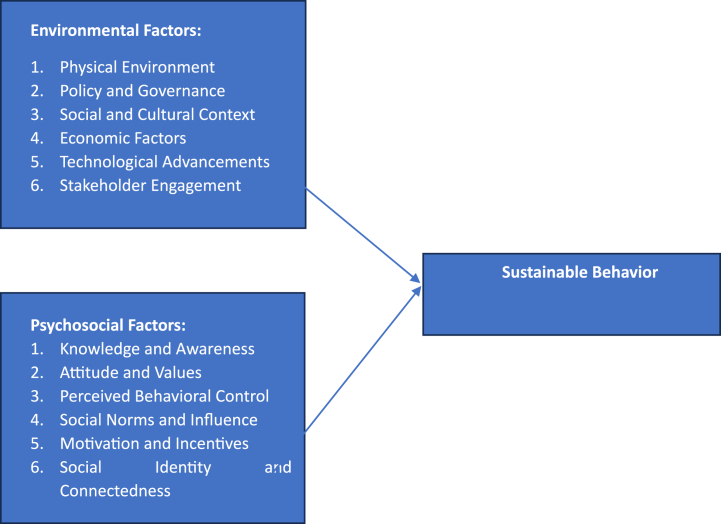
Conceptual framework.

Previous studies have consistently demonstrated a positive relationship between nature relatedness and environmental concern. Nisbet & Zelenski [18] found that individuals with a strong connection to nature tend to exhibit higher levels of environmental concern. Moreover, this concern for the environment has been shown to correlate positively with pro-environmental behavior, as indicated by studies conducted by Lange et al. [19] and Lange & Dewitte [20]. Gifford & Nilsson [21] and Wang et al. [22] further emphasize that individuals who care deeply for the natural environment are more inclined to take actions to protect it. This suggests that fostering nature relatedness may serve as a potential solution to current environmental challenges, as individuals who perceive themselves as integral parts of the Earth are more likely to engage in environmentally friendly behaviors [23].

Recognizing the importance of environmental concern in mitigating adverse effects on the environment, environmental education emerges as a crucial tool, particularly for younger generations [24] emphasize the significance of instilling environmental consciousness from an early age to empower future generations to address inherited environmental issues and prevent further damage. Indeed, research underscores the pivotal role of environmental education, especially for younger individuals, in promoting pro-environmental behavior [[25], [26], [27]]. Despite this, not everyone may prioritize environmental issues, as highlighted by Ref. [28]. Additionally, Grønhøj & Thøgersen [29] found that younger individuals may exhibit more reluctance towards pro-environmental behavior compared to their older counterparts. However, Liefländer et al. [30] suggest that strengthening connectedness to nature at an early age, ideally before the age of 11, can lead to more sustainable environmental attitudes and behaviors into adulthood. Therefore, integrating nature relatedness into school curricula emerges as a critical strategy to instill environmental concern among younger generations and foster a lifelong commitment to pro-environmental practices.

The importance of fostering a connection with nature from an early age to enhance environmental awareness and promote pro-environmental behavior stands out as a critical issue in previous research. Despite significant potential in environmental education, challenges such as generational differences, varying levels of engagement, and implementation barriers need to be carefully addressed to ensure that these efforts can have maximum impact in shaping a more sustainable future. Facing increasingly complex global environmental issues, it is crucial for educators, policymakers, and society to collaborate in creating inclusive and sustainable education strategies to ensure that the younger generation possesses the skills, knowledge, and attitudes necessary to preserve the planet for a better future.

This study seeks to address the research gap by focusing on the exploration of the psychological factors influencing sustainable behavior among children and adolescents in the school environment. By investigating the interplay between individual perceptions, motivations, attitudes, and values, and their impact on sustainable behavior, this research offers a novel perspective on the psychology of sustainability. It aims to provide insights into the underlying psychological processes that drive sustainable behavior among students, contributing to a more comprehensive understanding of sustainable practices within schools. Furthermore, this study takes a holistic approach by considering both the environmental and psychosocial factors that influence sustainable behavior.

This study delves into the exploration of Environmental and Psychosocial Factors impacting Sustainable Behavior among Indonesian adolescents, addressing a critical research gap. By examining how these factors operate within Indonesia's unique cultural and social milieu, the study aims to elucidate their influence on adolescents' decisions and actions concerning environmental sustainability. Consequently, it seeks to offer valuable insights into fostering sustainable behavior among Indonesian youth, guiding the development of effective educational strategies and interventions. Understanding the psychology of sustainability within the school environment is imperative in this context. Through investigating the interplay between environmental and psychosocial factors and sustainable behavior among students, the study endeavors to uncover areas for enhancement and formulate targeted strategies for promoting sustainable behavior. This deeper comprehension can inform the development of interventions and policies tailored to schools, nurturing sustainable values and attitudes among children and adolescents for a greener future. The research's significance lies in its potential to generate evidence-based interventions and strategies for cultivating sustainable behavior within schools. By pinpointing the psychological factors shaping sustainable behavior, educators and policymakers can craft interventions tailored to effectively instill sustainable values and practices among students. Ultimately, this research contributes to the burgeoning field of sustainable psychology, offering invaluable insights for fostering sustainable school environments and cultivating sustainable behaviors among the next generation.

## 1 Literature review

### 1.1 Context of the school education system in Indonesia

Indonesia has a complex and diverse education system, with a long history reflecting the cultural, social, and economic diversity present in the country. The education system in Indonesia consists of formal and non-formal education, with levels of education ranging from early childhood education to higher education. Education in Indonesia is regulated by the Ministry of Education, Culture, Research, and Technology (MoECRT), which is responsible for education policies and implementation nationwide. The school education system in Indonesia consists of three main levels: primary education, secondary education, and higher education. Primary education includes early childhood education (ECE) and 9-year basic education, while secondary education is divided into junior high school (SMP) and senior high school (SMA). Higher education includes universities, colleges, and other institutions of higher education. One of the main challenges in the school education system in Indonesia is the access and quality gap between urban and rural areas, as well as among the islands in the Indonesian archipelago. Additionally, the education system in Indonesia faces various issues, including a lack of adequate educational facilities, a shortage of qualified educators, and issues related to curriculum and teaching methods [31].

### 1.2 Relevance of environmental and sustainability issues

Environmental and sustainability issues are becoming increasingly important in the context of education in Indonesia [32]. With rapid economic growth and urbanization, Indonesia is faced with serious challenges related to environmental degradation, climate change, and the sustainability of natural resources. In the context of education, understanding environmental and sustainability issues is key to preparing future generations who are environmentally conscious and responsible. The involvement of schools in promoting environmental awareness and sustainable behavior is crucial in creating a culture of environmental care among young people [33]. Through integrated environmental education in the school curriculum, students can learn about the importance of preserving the environment, understand the impact of their actions on the environment, and develop the skills and attitudes necessary to behave sustainably.

### 1.3 Concept of sustainable behavior

Sustainable behavior is a crucial concept in achieving environmental and social sustainability goals [[34], [35], [36], [37]]. It encompasses the actions and decisions made by individuals and communities to maintain a balance between present needs and future requirements [38]. In the context of school environments, sustainable behavior includes environmentally-friendly practices such as the use of renewable energy, waste reduction, and awareness of environmental protection [39]. Several factors influence sustainable behavior. Firstly, environmental awareness plays a significant role in shaping individual behavior [40,41]. This involves understanding environmental issues such as climate change, biodiversity loss, and environmental degradation [42]. Secondly, individuals' attitudes towards the environment impact sustainable behavior [43,44]. Positive attitudes towards the environment encourage individuals to take more sustainable actions. Social norms also play a role in shaping sustainable behavior [45,46]. When individuals observe that sustainable behavior is supported by social norms within the school environment, they are more likely to follow and adopt such behaviors [47]. Additionally, individual motivation is a crucial driver of sustainable behavior [48]. Intrinsic motivations, such as a sense of ownership of the environment and personal satisfaction derived from sustainable actions, can propel individuals to engage in sustainable behavior [49]. Within the context of school environments, the role of the school environment itself as a shaping factor for sustainable behavior is crucial [50]. School environments that support sustainable practices, such as recycling facilities, environmental education programs, and student participation in environmental management, can foster sustainable behavior among children and adolescents. Conversely, school environments that do not support or even hinder sustainable practices may impede the development of sustainable behavior. In previous literature reviews, several studies have been conducted to understand the concept of sustainable behavior and the factors influencing it. Some studies highlight the importance of environmental education in school environments in shaping sustainable behavior [25,40,51]. Other studies emphasize the significant role of social norms in driving sustainable behavior [[52], [53], [54]], but limited research has been conducted on the perspective of the school environment in relation to this topic. Furthermore, some studies have focused on individual motivation [55,56] and economic factor [[57], [58], [59]] as a key factor in adopting sustainable behavior. However, despite the existing research, there are still research gaps that need to be addressed. One such gap pertains to the limited research specifically focusing on the concept of sustainable behavior in school environments and its relationship with psychosocial factors.

### 1.4 Environmental factors influencing sustainable behavior

Environmental factors play a significant role in influencing sustainable behavior among children and adolescents in the school environment [60]. These factors encompass various aspects that shape students' attitudes and actions towards sustainability. Firstly, the physical environment of the school, including infrastructure, facilities, and resources, can have a profound impact on sustainable behavior [[61], [62], [63]]. Access to recycling bins, energy-efficient systems, and green spaces can encourage environmentally conscious actions. Secondly, the rapid development and accessibility of technology have opened up new opportunities and avenues for promoting sustainability among students. Additionally, school policies and regulations play a crucial role in creating an enabling environment for sustainable behavior (T. [64]). Policies on waste management, energy conservation, water conservation, and sustainable transportation can provide clear guidelines and incentives for sustainable actions [65]. Furthermore, effective communication strategies and awareness campaigns can also contribute to sustainable behavior [66]. By disseminating information and organizing awareness events, students can develop a deeper understanding of the importance of sustainability and be motivated to adopt sustainable practices [67]. Moreover, involving students in collaborative projects, eco-clubs, and community engagement activities can foster a sense of ownership and commitment to sustainable behaviors [68]. By considering and addressing these environmental factors, educators and policymakers can create a supportive and inspiring school environment that promotes sustainable behavior among students, cultivating a generation that actively contributes to building a sustainable future [69]. Therefore, the hypothesis is.

H1a Physical Environment impact on Sustainable Behavior

H1b Policy and Governance impact on Sustainable Behavior

H1c Social and Cultural Context impact on Sustainable Behavior

H1d Economic Factors impact on Sustainable Behavior

H1e Technological Advancements impact on Sustainable Behavior

H1f Stakeholder Engagement impact on Sustainable Behavior

### 1.5 Psychosocial factors influencing sustainable behavior

Psychosocial factors play a crucial role in influencing sustainable behavior [[70], [71], [72]]. These factors encompass various psychological and social aspects that shape individuals' attitudes, beliefs, motivations, and values related to sustainability [50,73]. According to Shafiei & Maleksaeidi [74], individual perceptions of the importance of sustainability, their intrinsic motivations, and their sense of personal responsibility towards the environment can significantly impact their engagement in sustainable behaviors. Additionally, social influences such as peer norms, social support, and role models within the school community can shape students' attitudes and behaviors towards sustainability [75]. When sustainable behavior is positively reinforced and encouraged by peers and influential individuals, students are more likely to adopt and maintain sustainable practices [76]. The influence of educators, parents, and other significant adults in students' lives cannot be underestimated, as they play a crucial role in shaping their attitudes and values related to sustainability [77]. Furthermore, social attitude and value influence within the school community can significantly shape students' behavior [78]. Ephrem et al. [79] assert that when sustainable actions are perceived as socially desirable and accepted by peers, students are more likely to engage in them. By promoting environmental education, fostering a sense of environmental empathy and connectedness, and providing opportunities for active participation and meaningful engagement in sustainability-related activities, psychosocial factors can be leveraged to promote sustainable behavior among students in the school context [[80], [81], [82]]. Therefore, the hypothesis is.

H2a Knowledge and Awareness impact on Sustainable Behavior

H2b Attitudes and Values impact on Sustainable Behavior

H2c Perceived Behavioral Control impact on Sustainable Behavior

H2d Social Norms and Influence impact on Sustainable Behavior

H2e Motivation and Incentives impact on Sustainable Behavior

H2f Social Identity and Connectedness impact on Sustainable Behavior

## 2 Methodology

### 2.1 Research design, population, and sample

The study adopts a mixed-methods research design, integrating quantitative and qualitative methodologies. This combined approach facilitates a thorough exploration of the intricate connections among environmental factors, psychosocial factors, and sustainable behavior within the target demographic. The research population comprises students enrolled in different schools across Banten Province, Indonesia. Purposive sampling is employed to select a diverse and representative sample of participants meeting the study's inclusion criteria, which include considerations of age, gender, and socio-economic status of their families (see Table 1). The sample size is determined through statistical analysis to ensure adequate power for detecting significant relationships. In total, 159 participants from various high schools across Banten Province, Indonesia, are included in the study.

**Table 1:** Sample selection.

Categories	Value	Frequency (f)	f/total f (%)
Gender	Male	81	50.94
	Female	78	49.06
		**159**	**100.00**
Age	15	39	24.53
	16	41	25.79
	17	35	22.01
	18	44	27.67
		**159**	**100.00**
Socio-Economic Status	Low	46	28.93
	Medium	78	49.06
	High	35	22.01
		**159**	**100.00**
**Total**		**159**	**100.00**

The data presented in Table 1 illustrate the composition of the sample concerning gender, age, and socio-economic status. It reveals a balanced representation of participants across various demographics. Gender distribution shows a nearly equal split, with 81 males (50.94 %) and 78 females (49.06 %). Regarding age, participants are distributed quite evenly across different age groups, with the largest proportion being 18-year-olds (27.67 %), followed closely by 16-year-olds (25.79 %), 15-year-olds (24.53 %), and 17-year-olds (22.01 %). Furthermore, socio-economic status displays a varied distribution, with the majority coming from a middle socio-economic background (49.06 %), followed by those from low (28.93 %) and high (22.01 %) socio-economic statuses. Regarding low SES individuals have limited income, lower education, and low-skilled jobs with restricted access to services, medium SES individuals have moderate income and education with stable jobs, and high SES individuals enjoy high income, advanced education, and professional positions with full access to top-tier services. This diverse representation across gender, age, and socio-economic status categories enhances the robustness and generalizability of the study's findings.

### 2.2 Research variables

The independent variables in this study are environmental factors and psychosocial factors. Environmental factors encompass dimensions include the physical environment adopted from Lourenço et al. [83] (3 indicators), policy and governance adopted from T. Zhang et al. [64] (3 indicators), social and cultural context adopted from Sparkman et al. [52] (3 indicators), economic factors adopted from Nemeth et al. [57] (3 indicators), technological advancements adopted from Du et al. [1] (3 indicators), and stakeholder engagement adopted from Scurati et al. [81] (3 indicators). Psychosocial factors include knowledge and awareness adopted from Eugenio-Gozalbo et al. [84] and Maqsoom et al. [56] (3 indicators), attitudes and values adopted from Ayanwale et al. [78] (3 indicators), perceived behavioral control adopted from Caffaro et al. [70] (3 indicators), social norms and influence adopted from Ref. [46,54,79] (3 indicators), motivation and incentives adopted from Shafiei & Maleksaeidi [74] (3 indicators), and social identity and connectedness adopted from Siepelmeyer & Otterbring [46] (3 indicators). The dependent variable is sustainable behavior, which reflects the extent to which students engage in environmentally friendly practices use 8 indicators adopted from Ref. [14,61]. The indicators from the previous authors have been modified and adapted to suit the author's interests, with the purpose of simplifying the research to avoid delving into the context of educational sociology.

### 2.3 Data collection instruments, procedures, and analysis

In this study, a combination of quantitative and qualitative data collection instruments is utilized to comprehensively investigate the influence of environmental and psychosocial factors on sustainable behavior among students. Surveys, questionnaires, and scales are employed to gather quantitative data, while qualitative methods such as interviews and observations provide rich contextual insights. The data collection procedures involve administering surveys and questionnaires, conducting interviews, and making observations in the school environment (see Appendix 1). Stringent measures are implemented to safeguard participant privacy, ensure informed consent, and adhere to ethical standards throughout the data collection process. Following data collection, the quantitative data are subjected to confirmatory factor analysis (CFA) to validate the measurement models of the variables of interest. CFA assesses the reliability and validity of the measurement instruments used in the study. Furthermore, this study utilizes Path coefficient analysis, which is a statistical measure employed in path analysis or structural equation modeling to assess the strength and direction of relationships between variables within a model. The numerical values associated with path coefficients may vary depending on the model and data analyzed. It indicates how much change in the dependent variable is expected when the independent variable experiences a one-unit change, while controlling for other variables in the model. Path coefficients can provide information on the significance of the influence of independent variables on the dependent variable, as well as the direction of the relationship (positive or negative). This is a crucial measure in path analysis used to test hypotheses regarding the relationships between variables within a model.

Linguistic and translation issues play a critical role in research studies, particularly when data collection involves participants from diverse linguistic backgrounds. These issues are paramount as they can significantly affect the accuracy and reliability of the data gathered. One major concern is the accuracy of translations, as discrepancies or inaccuracies may lead to misunderstandings or misinterpretations of survey questions or interview prompts. Additionally, cultural sensitivity is essential, as certain words or phrases may carry different meanings or connotations across cultures. Without proper consideration, translated materials risk being culturally insensitive, potentially impacting participants' willingness to provide accurate responses. Ensuring equivalence between original and translated versions of data collection instruments is crucial to maintain consistency in measurement across different language versions. Furthermore, linguistic and translation issues can affect participants' comprehension of survey items or interview questions, potentially leading to response bias or misinterpretation of instructions. Addressing these issues is vital to uphold the quality and integrity of the data collected and to ensure that research findings accurately reflect the phenomena under investigation. Therefore, researchers must diligently address linguistic and translation issues to maintain the accuracy and validity of their research findings.

Subsequently, structural equation modeling (SEM) with the technique of Partial Least Squares (PLS) is employed using the Smart PLS application to analyze the relationships between the variables and test the hypothesized paths. PLS-SEM allows for the examination of complex causal relationships and provides insights into the direct and indirect effects of environmental and psychosocial factors on sustainable behavior. Additionally, qualitative data obtained from interviews and observations undergo thematic analysis to identify recurring patterns, themes, and underlying meanings. This qualitative approach complements the quantitative findings by providing deeper insights into the lived experiences and perspectives of the participants. Overall, this research methodology combines quantitative techniques such as confirmatory factor analysis and structural equation modeling with qualitative thematic analysis to offer a comprehensive understanding of the factors influencing sustainable behavior among students. By integrating multiple methodological approaches, the study contributes to advancing knowledge in this field and informing practical interventions for promoting sustainability in educational settings.

### 2.4 Ethics statement

This study was approved by the Ethics Committee of LPPM Paramadina University**.** Approval Number: 900/LPPM-UP/VI/2023. The study adhered to internationally recognized ethical standards as outlined in the Declaration of Helsinki and applicable national regulations. Moreover, all participants in this study provided written informed consent after receiving a comprehensive explanation of the study's purpose and methods, as well as potential risks and benefits. Confidentiality of personal information was assured, and participation was entirely voluntary.

## 3 Results

### 3.1 Validity and reliability

We assessed the validity of the indicator by employing the convergent method, which yielded the external loading factor. The acceptable range for the loading factor in exploratory studies, which are the initial stages of developing a measurement scale, is 0.50–0.70. In our specific investigation, all indicators exhibited an outer loading value greater than 0.70, meeting the criteria for convergent validity (refer to Table 1). In the next phase, we compared the square root coefficient of variance (AVE) extracted from each latent factor to the correlation coefficient between the other factors in the model. This analysis aimed to determine if the variables demonstrated discriminant validity, indicating their ability to differentiate between distinct groups. The AVE values significantly exceeded 0.5, as indicated in Table 1. Consequently, all constructs examined in this study exhibited discriminant validity higher than 0.50, as per Fornell & Larcker [85]. In the final step of the process, we employed composite reliability to assess the value of the variable indicators. Both the composite reliability and Cronbach's alpha exceeded 0.70, affirming the trustworthiness of the results [86].

The calculation of composite reliability for the variables in this study resulted in values ranging from 0.858 to 0.968, which exceeded the threshold of 0.70. These findings indicate that the indicators used to measure the variables were reliable and consistent. Additionally, Cronbach's alpha values ranging from 0.753 to 0.962 were obtained, further confirming the dependability of the indicators and indicating that they were free from measurement error [87].

### 3.2 Research findings

This study aims to examine the relationship between various environmental and psychosocial factors with sustainable behavior. The research methodology employed is a quantitative approach involving data collection through surveys or questionnaires. The constructs under investigation include physical environment, policy and governance, social and cultural context, economic factors, technological advancements, stakeholder engagement, knowledge and awareness, attitudes and values, perceived behavioral control, social norms and influence, motivation and incentives, as well as social identity and connectedness. Hypotheses have been formulated to test the relationship between each construct and sustainable behavior, which are then tested using T-statistics. The data analysis results indicate that all hypotheses are accepted, demonstrating a significant relationship between these factors and sustainable behavior.

The study found that environmental factors significantly impact students' sustainable behavior. Environmental factors such as supportive physical environments, sustainability-oriented policies and governance, and social and cultural contexts that promote sustainability values all play a role in shaping students' sustainable behavior. Students are more likely to engage in environmentally friendly practices when they are in a school environment that supports and encourages such behavior. The results of the study indicate that psychosocial factors also play a crucial role in shaping students' sustainable behavior. Positive attitudes and values related to sustainability, perceived behavioral control that instills confidence in individuals' ability to engage in sustainable actions, social norms and influence from the surrounding environment, motivation and incentives that encourage participation in sustainable practices, and social identity and connectedness to a community that cares about the environment are all associated with higher levels of sustainable behavior among students. Therefore, all hypotheses from H1a to H2f are accepted (see Table 2).

**Table 2:** Confirmatory factor analysis.

Construct	Items	Outer Loading	Cronbach's Alpha	rho_A	CR	AVE
Physical Environment	PE1 = Availability and accessibility of recycling facilities and waste management systems	0.812	0.822	0.837	0.893	0.736
	PE2 = Presence of green spaces, biodiversity, and natural resources	0.892				
	PE3 = Energy-efficient infrastructure and sustainable technologies within the environment	0.869				
Policy and Governance	PG1 = Existence of environmental policies, regulations, and incentives that promote sustainable practices	0.876	0.859	0.866	0.913	0.779
	PG2 = Integration of sustainability principles in educational curricula and school policies	0.881				
	PG3 = Collaboration between schools, local authorities, and organizations to implement sustainable initiatives	0.891				
Social and Cultural Context	SCC1 = Social norms and cultural values that prioritize environmental sustainability	0.891	0.823	0.834	0.894	0.738
	SCC2 = Supportive social networks and communities that encourage sustainable behaviors	0.851				
	SCC3 = Environmental education and awareness programs that promote sustainable values	0.835				
Economic Factors	EF1 = Affordability and accessibility of sustainable products and services	0.864				
	EF2 = Economic incentives for adopting sustainable practices, such as energy-saving measures or eco-friendly alternatives	0.862	0.845	0.848	0.906	0.763
	EF3= Integration of sustainability considerations in procurement and supply chain management	0.894				
Technological Advancements	TA1 = Availability and utilization of green technologies and innovations	0.947	0.879	0.877	0.927	0.810
	TA2 = Adoption of renewable energy sources and energy-efficient systems	0.800				
	TA3 = Integration of digital platforms and tools for monitoring and promoting sustainable behaviors	0.945				
Stakeholder Engagement	SE1 = Involvement of various stakeholders, such as students, teachers, parents, and community members, in sustainable initiatives	0.890	0.886	0.888	0.929	0.814
	SE2 = Collaboration between schools, businesses, and government agencies to foster sustainable practices	0.908				
	SE3 = Participation in environmental conservation and sustainability campaigns	0.910				
Knowledge and Awareness	KA1 = Knowledge about environmental issues and their impact on society	0.919	0.941	0.941	0.962	0.894
	KA2 = Awareness of sustainable practices and their benefits	0.96				
	KA3 = Understanding of the interconnectedness between human actions and the environment	0.958				
Attitudes and Values	AV1 = Positive attitudes towards environmental conservation and sustainability	0.840	0.908	0.945	0.942	0.844
	AV2 = Values that prioritize the well-being of the planet and future generations	0.947				
	AV3 = Belief in the importance of sustainable behavior for creating a better world	0.965				
Perceived Behavioral Control	PBC1 = Self-efficacy in engaging in sustainable actions	0.959	0.897	0.927	0.936	0.831
	PBC2 = Belief in personal agency to make a difference through individual behaviors	0.958				
	PBC3 = Confidence in one's ability to overcome barriers and challenges in practicing sustainability	0.809				
Social Norms and Influence	SN1 = Perception of social expectations regarding sustainable behavior.	0.854	0.906	0.907	0.941	0.843
	SN2 = Influence of peers, family, and community in promoting or discouraging sustainable actions	0.946				
	SN3 = Role models who exhibit sustainable behaviors and inspire others to follow suit	0.952				
Motivation and Incentives	MI1 = Intrinsic motivation to engage in sustainable behavior based on personal values and beliefs	0.797	0.753	0.76	0.858	0.668
	MI2 = Extrinsic incentives such as recognition, rewards, or social approval for sustainable actions	0.822				
	MI3 = Goal-setting and commitment to sustainability-related targets and initiatives	0.833				
Social Identity and Connectedness	SIC1 = Sense of belonging to a community that values sustainability	0.938	0.901	0.913	0.938	0.834
	SIC2 = Identification with environmental and sustainability-related groups or causes	0.941				
	SIC3 = Perceived social support for sustainable behavior	0.858				
Sustainable Behavior	SB1 = students' level of understanding and awareness of sustainability issues, including their knowledge of environmental challenges, social responsibility, and economic implications	0.864	0.962	0.966	0.968	0.793
	SB2 = students' behaviors related to waste reduction, recycling, and responsible waste management practices both at school and in their personal lives	0.892				
	SB3 = students' actions in conserving energy and natural resources, such as turning off lights when not in use, using energy-efficient devices, minimizing water consumption, and practicing responsible resource usage	0.918				
	SB4 = students' choices in transportation modes that have a lower environmental impact, such as walking, cycling, using public transportation, or carpooling	0.916				
	SB5 = students' purchasing decisions and consumption patterns, considering factors such as choosing environmentally friendly products, reducing single-use items, and practicing conscious and responsible consumption	0.926				
	SB6 = students' involvement in sustainability-related activities, such as participating in environmental clubs or initiatives, advocating for sustainable policies, and engaging in community service projects focused on sustainability	0.906				
	SB7 = students' sense of responsibility and active involvement in protecting and preserving the natural environment, including activities such as planting trees, participating in ecological restoration projects, and promoting biodiversity conservation	0.792				
	SB8 = students' ability to work collaboratively with peers, teachers, and other stakeholders to address sustainability challenges and implement sustainable initiatives within the school community	0.903				

The Path Coefficient table (Table 3) presents the results of the analysis conducted to examine the relationship between various constructs and sustainable behavior. Each row represents a hypothesis tested, indicating the construct being examined in relation to sustainable behavior, the original sample coefficient, t-statistics, p-values, and the remark based on the analysis. The analysis reveals significant relationships between different constructs and sustainable behavior. For instance, concerning the impact of environmental factors, the coefficient for the relationship between the Physical Environment and Sustainable Behavior (H1a) is 0.621, indicating a moderate positive association. Similarly, constructs such as Policy and Governance (H1b) is 0.481, Social and Cultural Context (H1c) is 0.437, Economic Factors (H1d) is 0.671, Technological Advancement (H1e) is 0.709, Stakeholder Engagement (H1f) is 0.665, Knowledge and Awareness (H2a) is 0.489, Attitudes and Values (H2b) is 0.458, Perceived Behavioral Control (H2c) is 0.653, Social Norms and Influence (H2d) is 0.668, Motivation and Incentives (H2e) is 0.525, and Social Identity and Connectedness (H2f) is 0.701 also show significant positive associations with sustainable behavior, as evidenced by their respective coefficients and statistical values.

**Table 3:** Path coefficient.

Hypothesis	Construct *)	Original Sample	T Statistics	P Values	Remark
H1a	Physical Environment - > Sustainable Behavior	0.621	2.573	0.000	Proven
H1b	Policy and Governance - > Sustainable Behavior	0.481	2.235	0.000	Proven
H1c	Social and Cultural Context - > Sustainable Behavior	0.437	2.214	0.000	Proven
H1d	Economic Factors - > Sustainable Behavior	0.671	2.977	0.000	Proven
H1e	Technological Advancements - > Sustainable Behavior	0.709	5.848	0.000	Proven
H1f	Stakeholder Engagement - > Sustainable Behavior	0.665	2.583	0.000	Proven
H2a	Knowledge and Awareness - > Sustainable Behavior	0.489	2.239	0.000	Proven
H2b	Attitudes and Values - > Sustainable Behavior	0.458	2.232	0.000	Proven
H2c	Perceived Behavioral Control - > Sustainable Behavior	0.653	2.505	0.000	Proven
H2d	Social Norms and Influence - > Sustainable Behavior	0.668	2.549	0.000	Proven
H2e	Motivation and Incentives - > Sustainable Behavior	0.525	2.636	0.000	Proven
H2f	Social Identity and Connectedness - > Sustainable Behavior	0.701	5.828	0.000	Proven

Moreover, the t-statistics for each relationship are notably high, indicating the robustness and reliability of the observed associations. Additionally, all p-values are recorded as 0.000, signifying a high level of statistical significance. Consequently, based on the analysis, all hypotheses tested are confirmed, as indicated by the “Proven” remark in the table. Overall, the findings from the Path Coefficient table underscore the importance of various factors, including environmental, social, cultural, economic, and psychological aspects, in influencing sustainable behavior. These results provide valuable insights for policymakers, educators, and stakeholders in designing effective interventions and strategies to promote sustainable behavior within communities and organizations.

## 4 Discussion

### 4.1 Environmental factors influencing sustainable behavior

Acceptance of hypothesis 1 (H1a) implies that the physical school environment plays a crucial role in influencing students' sustainable behavior. It serves as a tangible and visible setting where students learn, interact, and develop their values and behaviors. Lourenço et al. [83] assert that the design and features of the school buildings, classrooms, outdoor spaces, and facilities significantly impact students' perceptions and actions towards sustainability. For instance, well-designed waste management infrastructure, such as recycling bins and composting systems, can encourage students to practice proper waste segregation and recycling. Energy-efficient measures, such as energy-saving lighting and smart thermostats, can raise awareness about energy conservation and motivate students to adopt energy-saving habits. The presence of green spaces and gardens provides opportunities for students to connect with nature, develop an appreciation for the environment, and learn about sustainable practices like organic farming and biodiversity conservation. Furthermore, promoting sustainable transportation options and designing eco-friendly buildings with natural lighting and sustainable materials can inspire students to make environmentally friendly choices and understand the importance of sustainable living. By creating an environmentally conscious physical school environment, educational institutions can shape students' behaviors, attitudes, and values towards sustainability, fostering a generation of environmentally responsible individuals [[88], [89], [90]].

The acceptance of hypothesis 2 (H1b) implies that there is a positive evaluation of the validity or truthfulness of the hypothesis. In this context, if hypothesis 2 (H1b) is accepted, it indicates that there is evidence or findings that support the hypothesis that Policies and governance play a crucial role in shaping sustainable behavior. In the context of sustainable behavior, this finding aligns with the perspective of T. Zhang et al. [64], who suggested that supportive policies and effective governance provide a solid framework for individuals and communities to adopt sustainable practices. Furthermore, these findings corroborate the views expressed by Naidoo & Gasparatos [65] that policies can include environmental regulations, energy policies, waste management policies, and economic incentives to encourage sustainable actions. For instance, school policies that prohibit the use of single-use plastics or mandate recycling programs can influence students' behavior in plastic usage and reduce waste generation. Additionally, strong and effective governance in the context of sustainable education involves the active participation of all stakeholders, including teachers, students, school staff, parents, and the local community. Through dialogue and collaboration, sound policies and governance can create a supportive environment for developing awareness, understanding, and commitment to sustainable behavior. With adequate policies and governance, schools can become institutions that teach and practice sustainable values, fostering a culture that encourages and facilitates the implementation of sustainable behavior in the daily lives of students and the surrounding community.

In the context of sustainable behavior, social and cultural factors play a significant role in shaping students' sustainable behavior. This provides evidence that hypothesis 3 (H1c) is accepted, indicating the importance of social and cultural factors in influencing sustainable behavior. These findings further confirm the research outcomes of Sparkman et al. [52], that the social and cultural environment in which students grow and learn has the power to shape values, norms, and mindsets associated with sustainability. On the other hand, this finding aligns with the perspective of Vögele et al. [53], that social values that support sustainability, such as responsibility towards the environment, cooperation, and awareness of individual impact on the environment, can influence students' behavior in adopting sustainable actions. Additionally, cultural factors also have an important role. Furthermore, these findings once again substantiate the explanations provided by Yamin et al. [54], that culture can influence students' perceptions, preferences, and habits related to sustainability. For example, in cultures where respect for nature and sustainability are integral parts of societal values, students are more likely to have a higher awareness of the importance of sustainable behavior. Moreover, social norms also play a significant role. If sustainable behavior is perceived as a norm accepted by the majority of individuals in the social environment of students, they are more likely to adopt such behavior. A supportive social environment that encourages and promotes sustainable behavior, whether through peer examples or encouragement from family and community, can influence students to internalize these values and norms. In this context, it is important for education to consider the social and cultural dimensions in developing sustainable education programs. Identifying and understanding the social and cultural factors influencing students' sustainable behavior can help design more effective strategies to promote awareness, understanding, and acceptance of sustainability values within the school community. By creating a supportive social and cultural environment, students can actively engage in sustainable practices and drive sustainable behavior change within themselves and the communities they reside in.

Economic factors play a significant role in influencing students' sustainable behavior. This provides evidence that hypothesis 4 (H1d) is accepted, indicating the importance of economic factors in influencing sustainable behavior. This finding in line with the views of Thieme et al. [58] that the economic context in which students live and study can shape their attitudes, choices, and actions towards sustainability. Economic factors such as the cost of living, availability and affordability of sustainable products, and financial constraints can impact students' ability to adopt sustainable behaviors. For instance, if sustainable options are perceived as more expensive or financially burdensome, students may be less likely to prioritize them over more affordable alternatives. Additionally, this finding is in line with the perspective of Yalin et al. [59] that economic incentives and disincentives, such as scholarships, grants, or financial rewards for sustainable practices, can motivate students to engage in sustainable behaviors. Furthermore, it finding in line with the views of Nemeth et al. [57] that economic considerations are also relevant in educational settings. Schools and universities that allocate resources towards sustainable initiatives, such as energy-efficient buildings or waste reduction programs, create an environment that promotes and supports sustainable behavior among students. Moreover, economic factors can influence the broader socio-economic conditions that students face, including access to education, employment opportunities, and social support systems, all of which can influence their ability to engage in sustainable practices. Therefore, addressing economic factors is crucial in fostering student's sustainable behavior by ensuring the affordability and accessibility of sustainable options, providing economic incentives for sustainable actions, and creating an inclusive socio-economic environment that supports sustainable practices among students.

Hypothesis 5 (H1e) is accepted, providing evidence that technological advancements have a profound influence on students' sustainable behavior. The rapid development and accessibility of technology have opened up new opportunities and avenues for promoting sustainability among students. Technology provides innovative solutions and tools that can empower students to adopt and engage in sustainable practices. For instance, this finding is in accordance with the views of Abbas et al. [61] that digital platforms and applications can offer educational resources, interactive learning experiences, and gamification elements that enhance students' understanding of sustainability and motivate them to take sustainable actions. Furthermore, this finding also supports the viewpoint of Du et al. [1] that technological advancements enable the implementation of smart systems and energy-efficient infrastructure in educational institutions, creating environmentally friendly learning environments that inspire sustainable behaviors. Additionally, the finding of this study further corroborate the study by Zhou et al. [5] which suggests that technology facilitates communication and collaboration among students, allowing them to share ideas, knowledge, and initiatives related to sustainability. Social media platforms and online communities provide spaces for students to connect, learn from one another, and collectively advocate for sustainable causes. Moreover, it finding in line with the views of Abbas et al. [61] and Scurati et al. [81] that technology plays a significant role in data collection, analysis, and monitoring of sustainable behaviors, enabling educational institutions to track and assess students' progress towards sustainability goals. However, it is essential to ensure equitable access to technology to avoid creating a digital divide that may hinder some students' participation in sustainable initiatives. Overall, technological advancements offer immense potential in driving student's sustainable behavior by providing educational resources, enabling energy-efficient infrastructure, fostering collaboration, and facilitating data-driven approaches to sustainability.

Stakeholder engagement plays a crucial role in influencing students' sustainable behavior. It provides evidence that hypothesis 6 (H1f) is accepted, indicating the importance of stakeholder engagement in influencing sustainable behavior. This finding serves as evidence supporting the study by Aleixo et al. [67] that the active involvement of various stakeholders, including educators, parents, community members, and policymakers, creates a supportive ecosystem that fosters sustainable practices among students. When stakeholders collaborate and align their efforts towards sustainability, they can collectively contribute to creating an environment that promotes and reinforces sustainable behavior. Educators play a key role in integrating sustainability into the curriculum, teaching students about the importance of environmental conservation, and empowering them with the knowledge and skills to make sustainable choices. Parents and families also play a significant role by instilling sustainable values and behaviors at home and encouraging their children to participate in sustainability initiatives. Engaging community members, local organizations, and businesses further expands the scope of sustainability education by providing students with real-world examples and opportunities to apply their knowledge. Additionally, these results provide evidence for the viewpoint expressed by Oe et al. [12] that policymakers play a vital role in creating supportive policies and frameworks that prioritize sustainability in educational institutions and the broader community. These results are consistent with the explanation provided by Scurati et al. [81] that by involving stakeholders in decision-making processes and fostering collaboration, students are more likely to internalize sustainable values and behaviors and become active contributors to a sustainable future. Furthermore, stakeholder engagement can lead to the establishment of partnerships and networks that enhance access to resources, funding, and expertise, enabling the implementation of sustainable initiatives in schools. Overall, stakeholder engagement creates a dynamic and interconnected environment that empowers students to embrace sustainable behaviors through comprehensive support, collaboration, and shared responsibility.

### 4.2 Psychosocial factors influencing sustainable behavior

Hypothesis 7 (H2a) is accepted, providing evidence that knowledge and awareness play a crucial role in shaping individuals' sustainable behavior. In the context of students, in line with the findings of Maqsoom et al. [56] that having a strong foundation of knowledge about environmental issues, sustainability concepts, and their implications is essential in fostering sustainable behavior. When students are equipped with the necessary knowledge, they can understand the interconnections between their actions and the environment, and recognize the importance of making sustainable choices. Awareness, on the other hand, involves a deep understanding and consciousness of the impact of individual actions on the environment and society. By raising awareness among students about sustainability challenges and the benefits of sustainable practices, they can develop a sense of responsibility and motivation to adopt sustainable behaviors. Once again, this study provides further evidence supporting the findings of Eugenio-Gozalbo et al. [84] that education plays a vital role in enhancing knowledge and awareness, both within formal classroom settings and through informal learning opportunities. Incorporating sustainability into the curriculum, organizing awareness campaigns, and providing experiential learning opportunities such as field trips and environmental projects are effective strategies to enhance students' knowledge and awareness. Additionally, leveraging technology and digital platforms can broaden access to information and resources, allowing students to stay informed and engaged in sustainability issues. Furthermore, fostering a culture of critical thinking, inquiry, and open dialogue encourages students to question existing norms and explore innovative solutions for sustainability challenges. Ultimately, by promoting knowledge and awareness among students, we can empower them to make informed choices, take responsible actions, and become agents of positive change towards a more sustainable future.

The acceptance of hypothesis 8 (H2b) serves as evidence that attitudes and values play a significant role in influencing students' sustainable behavior. It finding proves the viewpoint of H. Zhang & Gibson [8] and Braun et al. [88] that attitudes reflect individuals' evaluations and feelings towards sustainability-related issues, while values represent deeply held beliefs and principles. This result also validate the viewpoint of Ayanwale et al. [78] assert that positive attitudes towards sustainability, such as recognizing its importance and benefits, are essential in motivating students to engage in sustainable behaviors. When students perceive sustainability as personally relevant and meaningful, they are more likely to adopt and maintain sustainable practices. Moreover, values, such as environmental stewardship, social responsibility, and intergenerational equity, guide students' decision-making processes and shape their long-term commitment to sustainable behavior. When sustainability aligns with students' core values, it becomes an integral part of their identity and guides their actions in various contexts. Creating a supportive school environment that nurtures positive attitudes and values towards sustainability is crucial. This can be achieved through educational programs, extracurricular activities, and the integration of sustainability principles into school policies and practices. Role modeling by teachers and school staff also plays a vital role in influencing students' attitudes and values. Through the cultivation of a sustainability-oriented culture and the encouragement of favorable attitudes towards it, educational institutions have the potential to play a pivotal role in nurturing individuals who are environmentally aware and socially accountable. By instilling values that prioritize sustainability and fostering positive perceptions regarding its significance, schools can contribute to the formation of conscientious individuals who actively engage in activities aimed at achieving a more sustainable future.

Hypothesis 9 (H2c) is also accepted, providing evidence that perceived behavioral control is a significant factor that relates to students' sustainable behavior. It refers to individuals' beliefs about their ability to perform a particular behavior and exert control over the outcomes. In the context of sustainability, This serves as proof from the study conducted by Ref. [70]Caffaro et al. [70] and de Leeuw et al. [16] that perceived behavioral control encompasses students' confidence in their capacity to engage in sustainable actions and their perception of the resources and opportunities available to support such behaviors. When students have a high level of perceived behavioral control, they are more likely to believe that their actions can make a difference in promoting sustainability and that they have the necessary skills and resources to overcome barriers and challenges. This sense of control empowers students to take proactive steps towards sustainable behavior, even in the face of external constraints or social pressures. Educational interventions that enhance students' self-efficacy, provide them with the necessary knowledge and skills, and create supportive environments can effectively strengthen perceived behavioral control and promote sustainable behavior.

Social norms and influence play a crucial role in shaping students' sustainable behavior. It provides evidence that hypothesis 10 (H2d) is accepted, indicating the importance of social norms in influencing sustainable behavior. As social beings, individuals are influenced by the norms and expectations of their social groups, including peers, teachers, and the wider community. When sustainable behavior is widely accepted and encouraged within these social contexts, it creates a normative influence that can motivate students to adopt and engage in sustainable practices. Additionally, social influence mechanisms such as peer pressure, social approval, and role modeling can further reinforce sustainable behaviors among students. By promoting positive social norms and fostering a supportive social environment, schools can harness the power of social influence to encourage and sustain students' engagement in sustainable behavior. The results provide evidence that supports the previous study's findings that social norms have an influence on behavior [46,54,79].

Motivation and incentives play a significant role in influencing students' sustainable behavior. This is demonstrated by the acceptance of hypothesis 11 (H2e) that motivation and incentives have a positive and significant influence on sustainable behavior. Motivation refers to the internal drives, desires, and goals that direct individuals' behavior. When students are intrinsically motivated to engage in sustainable practices, such as a genuine concern for the environment or a sense of personal responsibility, they are more likely to adopt and maintain sustainable behaviors. Additionally, extrinsic motivation in the form of incentives can further enhance students' engagement in sustainable behavior. Incentives, such as rewards, recognition, or tangible benefits, provide external reinforcement and can serve as powerful motivators for students to actively participate in sustainable initiatives. By understanding the factors that motivate students and designing effective incentive systems, schools can create an environment that nurtures and sustains students' motivation to practice and promote sustainable behaviors. The findings offer evidence that corroborates the earlier study's conclusions that motivation and incentives play a role in shaping behavior [46,74].

In the context of sustainable behavior, social identity and connectedness have a strong influence on students' sustainable behavior. This provides evidence that hypothesis 12 (H2f) is accepted, indicating the importance of social and cultural factors in influencing sustainable behavior. It finding align with the standpoint of Siepelmeyer & Otterbring [46] that social identity refers to the sense of belonging and identification individuals feel with a particular social group, such as their school community or environmental clubs. When students identify themselves as members of a community that values sustainability, they are more likely to adopt sustainable behaviors as a way to align with the group's norms and values. Moreover, connectedness, which refers to the sense of interpersonal connection and support, plays a crucial role in promoting sustainable behavior. When students feel connected to their peers, teachers, and the wider community, they are more likely to engage in collaborative sustainability efforts and seek collective solutions to environmental challenges. Creating opportunities for students to develop a strong social identity and fostering a sense of connectedness within the school environment can effectively promote sustainable behavior by harnessing the power of social influence and collective action.

### 4.3 The socio-economic and cultural aspects of Indonesia

The data analysis indicates that all hypotheses tested in this study are statistically significant, with p-values lower than 0.05. This suggests that the constructs examined, ranging from the physical environment to social identity and connectedness, have a significant relationship with sustainable behavior in Indonesia. The interpretation of these findings strengthens our understanding of the crucial role various aspects play in influencing individuals' attitudes and actions towards sustainability. In the economic context, the findings highlight the highly influential role of economic factors in shaping sustainable behavior in Indonesia. For example, socio-economic conditions such as income levels, accessibility to healthcare services, and unemployment rates can influence individuals' ability to adopt sustainable behaviors. In Indonesia, where economic disparities between urban and rural areas are still significant, differences in access to resources and infrastructure also play a crucial role in determining the level of participation in sustainable practices.

Additionally, cultural aspects also have a significant impact on sustainable behavior. Cultural values such as mutual cooperation (gotong royong) and the rich cultural diversity in Indonesia can motivate individuals to take actions that support environmental sustainability. Moreover, local traditions such as traditional ceremonies and religious practices often have components that promote environmental awareness and social responsibility. Overall, understanding the complexity of interactions between specific socio-economic and cultural factors in Indonesia can provide valuable insights into developing effective strategies and policies to promote sustainable behavior in society.

## 5 Conclusions

The research delves into the intricate dynamics influencing sustainable behavior among students in Indonesia, uncovering a rich tapestry of interconnected factors. Central to this analysis is the pivotal role of the physical school environment, which serves as a tangible locus for shaping sustainable practices. From well-designed waste management systems to energy-efficient infrastructure and green spaces, the physical setting of schools lays the foundation for instilling environmental consciousness among students. Furthermore, the study underscores the significance of supportive policies and governance frameworks in fostering sustainable behavior. By enacting regulations and incentives, educational institutions and policymakers can create an environment that not only encourages but also regulates sustainable actions, aligning institutional objectives with broader societal goals of environmental stewardship. In addition to the institutional framework, social and cultural factors emerge as influential drivers of sustainable behavior. The research highlights the profound impact of societal norms, values, and cultural practices on students' attitudes and actions towards sustainability. Within the social milieu of Indonesian society, where respect for nature and collective responsibility often hold sway, students are imbued with a sense of duty towards environmental conservation. Moreover, economic considerations play a significant role in shaping sustainable behavior, with accessibility and affordability of eco-friendly alternatives influencing consumption patterns.

The study underscores the need for targeted interventions that address economic disparities and provide incentives for sustainable choices, ensuring that financial constraints do not hinder the adoption of environmentally friendly practices among students. Furthermore, the study underscores the transformative potential of technology in driving sustainable behavior change among students. From digital platforms offering educational resources to smart systems facilitating energy efficiency, technological innovations offer a myriad of opportunities to engage and empower students in sustainability initiatives. By harnessing the power of technology, educational institutions can bridge knowledge gaps, foster collaboration, and facilitate data-driven approaches to sustainability. However, equitable access to technology remains paramount to ensure that all students can benefit from these advancements, underscoring the need for inclusive policies that address digital divides and promote equal opportunities for participation in sustainable initiatives.

The research findings have significant implications for the development of sustainable school environments. By understanding the influence of environmental and psychosocial factors on students' sustainable behavior, schools can design and implement strategies that create a supportive and conducive environment for sustainability. This includes incorporating sustainable practices into the physical infrastructure of the school, such as energy-efficient systems, waste management strategies, and eco-friendly facilities. Additionally, promoting a culture of sustainability through curriculum integration, extracurricular activities, and community engagement can reinforce sustainable values and behaviors among students. The findings emphasize the importance of considering the school environment as a critical component in fostering sustainable behavior among students. The research findings provide valuable insights for the development of interventions and policies in schools aimed at promoting sustainable behavior among students. Educators and policymakers can use the knowledge gained from this research to design targeted interventions that address the specific dimensions and factors influencing sustainable behavior. This may involve implementing educational programs that enhance students' knowledge and awareness, promoting positive attitudes and values through curriculum development, and providing incentives and motivation for sustainable practices. Furthermore, the findings highlight the need for comprehensive policies that integrate sustainability into various aspects of school life, including curriculum, infrastructure, and school governance. By aligning interventions and policies with the research findings, schools can effectively promote sustainable behavior among students.

While the study offers valuable insights into the psychological factors influencing sustainable behavior within the school context in the province of Banten and Indonesia at large, it is essential to acknowledge its limitations. Firstly, the reliance on self-reported data introduces the possibility of response bias and social desirability effects, potentially influencing the accuracy of the findings. Additionally, the study's focus on a specific geographical area and school setting may limit the generalizability of the results to broader contexts. Variations in socio-cultural norms, economic conditions, and educational policies across different regions could impact the applicability of the findings beyond the study's scope.

Moreover, the cross-sectional nature of the research design restricts the ability to establish causal relationships between variables. Longitudinal studies could provide a deeper understanding of how psychological factors evolve over time and their impact on sustainable behavior outcomes. Furthermore, while the study addresses a significant research gap by emphasizing psychological aspects of sustainability in schools, future research could explore additional contextual factors that may influence sustainable behavior, such as community characteristics, governance structures, and economic conditions. Despite these limitations, the study's strengths, including the use of diverse data collection methods and the inclusion of a wide range of participants, contribute to its significance and reliability. By leveraging these strengths and addressing the identified limitations, future research can build upon the study's findings to develop more robust interventions, policies, and practices aimed at promoting sustainable behavior in schools. Ultimately, a concerted effort by educators, policymakers, and researchers is necessary to create environmentally conscious school environments that empower students to embrace sustainability and contribute to a more sustainable future.

## Data availability statement

The data that support the findings of this study are available from the corresponding author upon reasonable request.

## Ethical statement and approval

I, the undersigned, Tia Rahmania, as in the name of chairperson of the Ethics Committee of LPPM Paramadina University, hereby declare that the research proposal submitted by Tia Rahmania has received approval from our Ethics Committee with approval number 900/LPPM-UP/VI/2023. All procedures proposed in this study have been reviewed and approved for compliance with applicable ethical guidelines and the Declaration of Helsinki. Participants in this research are guaranteed to receive adequate information regarding the research objectives, procedures, risks, and benefits, and will provide written informed consent before participating. We ensure that participant data will be kept confidential and used exclusively for the purposes of this research.

## CRediT authorship contribution statement

**Tia Rahmania:** Writing – review & editing, Writing – original draft, Validation, Software, Methodology, Investigation, Funding acquisition, Formal analysis, Data curation, Conceptualization.

## Declaration of competing interest

The authors declare the following financial interests/personal relationships which may be considered as potential competing interests:Tia Rahmania reports was provided by Paramadina University. Tia Rahmania reports a relationship with University of Paramadina that includes: The author, Tia Rahmania, declares that I am a member of the ethics committee that provided approval for this study. The approval process also involved Cut Mellyza Rizka as the chair of the ethics committee. There are no other conflicts of interest to declare. If there are other authors, they declare that they have no known competing financial interests or personal relationships that could have appeared to influence the work reported in this paper.
