# Supplementary material for: Exploring school environmental psychology in children and adolescents: The influence of environmental and psychosocial factors on sustainable behavior in Indonesia
Source: Heliyon. 2024 Sep 12;10(18):e37881. doi: 10.1016/j.heliyon.2024.e37881 (PMC11417535; doi:10.1016/j.heliyon.2024.e37881)
Supplement: Multimedia component 2 [file mmc2.docx]

**Dear [Participant's Name],**

We are conducting a study to understand the various factors that influence sustainable behavior among students. Your valuable input is crucial in helping us gain insights into this important area. Please take a few minutes to answer the following questions. Your responses will be kept confidential and used solely for research purposes.

**Physical Environment:**

PE1. How easily accessible are recycling facilities and waste management systems at your school? (1) Very Inaccessible - (5) Very Accessible

PE2. Do you believe your school has enough green spaces, biodiversity, and natural resources? (1) Strongly Disagree - (5) Strongly Agree

PE3. Are there energy-efficient infrastructure and sustainable technologies implemented within your school environment? (1) Not at All - (5) Fully Implemented

**Policy and Governance:**

PG1. Are there environmental policies and incentives in place to promote sustainable practices at your school? (1) No - (5) Yes, extensively

PG2. To what extent do you think sustainability principles are integrated into your school's educational curricula and policies? (1) Not Integrated - (5) Fully Integrated

PG3. Are there collaborative efforts between your school, local authorities, and organizations to implement sustainable initiatives? (1) Very Few - (5) Extensive Collaboration

**Social and Cultural Context:**

SCC1. Do you perceive that social norms and cultural values in your school prioritize environmental sustainability? (1) Strongly Disagree - (5) Strongly Agree

SCC2. Are there supportive social networks and communities within your school that encourage sustainable behaviors? (1) None - (5) Strong Support

SCC3. Have you been involved in environmental education and awareness programs at your school? (1) Not at All - (5) Extensively Involved

**Economic Factors:**

EF1. Are sustainable products and services affordable and accessible at your school? (1) Not Accessible - (5) Highly Accessible

EF2. Are there economic incentives for adopting sustainable practices at your school? (1) None - (5) Significant Incentives

EF3. Is sustainability considered in procurement and supply chain management within your school? (1) Not Considered - (5) Fully Integrated

Your participation in this survey is greatly appreciated, and your responses will contribute to our understanding of promoting sustainability in educational environments.

Thank you for your time.

Sincerely,

Tia Rahmania
