# Supplementary material for: Exploring school environmental psychology in children and adolescents: The influence of environmental and psychosocial factors on sustainable behavior in Indonesia
Source: Heliyon. 2024 Sep 12;10(18):e37881. doi: 10.1016/j.heliyon.2024.e37881 (PMC11417535; doi:10.1016/j.heliyon.2024.e37881)
Supplement: Multimedia component 3 [file mmc3.docx]

Appendix 1

**Questionnaire**

1. Availability and accessibility of recycling facilities and waste management systems in my school are adequate.

- PE1:

(1) Strongly Disagree

(2) Disagree

(3) Neutral

(4) Agree

(5) Strongly Agree

2. The presence of green spaces, biodiversity, and natural resources in my school is noticeable.

- PE2:

(1) Strongly Disagree

(2) Disagree

(3) Neutral

(4) Agree

(5) Strongly Agree

3. My school has energy-efficient infrastructure and sustainable technologies incorporated into the environment.

- PE3:

(1) Strongly Disagree

(2) Disagree

(3) Neutral

(4) Agree

(5) Strongly Agree

4. My school has environmental policies, regulations, and incentives in place that promote sustainable practices.

- PG1:

(1) Strongly Disagree

(2) Disagree

(3) Neutral

(4) Agree

(5) Strongly Agree

5. Sustainability principles are integrated into the educational curricula and school policies.

- PG2:

(1) Strongly Disagree

(2) Disagree

(3) Neutral

(4) Agree

(5) Strongly Agree

6. There is collaboration between my school, local authorities, and organizations to implement sustainable initiatives.

- PG3:

(1) Strongly Disagree

(2) Disagree

(3) Neutral

(4) Agree

(5) Strongly Agree

7. Social norms and cultural values in my school prioritize environmental sustainability.

- SCC1:

(1) Strongly Disagree

(2) Disagree

(3) Neutral

(4) Agree

(5) Strongly Agree

8. My school fosters supportive social networks and communities that encourage sustainable behaviors.

- SCC2:

(1) Strongly Disagree

(2) Disagree

(3) Neutral

(4) Agree

(5) Strongly Agree

9. Environmental education and awareness programs in my school effectively promote sustainable values.

- SCC3:

(1) Strongly Disagree

(2) Disagree

(3) Neutral

(4) Agree

(5) Strongly Agree

10. The affordability and accessibility of sustainable products and services are prioritized in my school.

- EF1:

(1) Strongly Disagree

(2) Disagree

(3) Neutral

(4) Agree

(5) Strongly Agree

11. There are economic incentives in place at my school to encourage the adoption of sustainable practices, such as energy-saving measures or eco-friendly alternatives.

- EF2:

(1) Strongly Disagree

(2) Disagree

(3) Neutral

(4) Agree

(5) Strongly Agree

12. Sustainability considerations are integrated into procurement and supply chain management processes at my school.

- EF3:

(1) Strongly Disagree

(2) Disagree

(3) Neutral

(4) Agree

(5) Strongly Agree

13. My school has access to and effectively utilizes green technologies and innovations.

- TA1:

(1) Strongly Disagree

(2) Disagree

(3) Neutral

(4) Agree

(5) Strongly Agree

14. Renewable energy sources and energy-efficient systems are actively adopted and implemented in my school.

- TA2:

(1) Strongly Disagree

(2) Disagree

(3) Neutral

(4) Agree

(5) Strongly Agree

15. Digital platforms and tools are integrated into my school for monitoring and promoting sustainable behaviors.

- TA3:

(1) Strongly Disagree

(2) Disagree

(3) Neutral

(4) Agree

(5) Strongly Agree

16. Various stakeholders, such as students, teachers, parents, and community members, are actively involved in sustainable initiatives at my school.

- SE1:

(1) Strongly Disagree

(2) Disagree

(3) Neutral

(4) Agree

(5) Strongly Agree

17. Collaboration between my school, businesses, and government agencies is established to foster sustainable practices.

- SE2:

(1) Strongly Disagree

(2) Disagree

(3) Neutral

(4) Agree

(5) Strongly Agree

18. My school actively participates in environmental conservation and sustainability campaigns.

- SE3:

(1) Strongly Disagree

(2) Disagree

(3) Neutral

(4) Agree

(5) Strongly Agree

19. I possess knowledge about environmental issues and their impact on society.

- KA1:

(1) Strongly Disagree

(2) Disagree

(3) Neutral

(4) Agree

(5) Strongly Agree

20. I am aware of sustainable practices and their benefits.

- KA2:

(1) Strongly Disagree

(2) Disagree

(3) Neutral

(4) Agree

(5) Strongly Agree

21. I understand the interconnectedness between human actions and the environment.

- KA3:

(1) Strongly Disagree

(2) Disagree

(3) Neutral

(4) Agree

(5) Strongly Agree

22. I hold positive attitudes towards environmental conservation and sustainability.

- AV1:

(1) Strongly Disagree

(2) Disagree

(3) Neutral

(4) Agree

(5) Strongly Agree

23. I prioritize the well-being of the planet and future generations.

- AV2:

(1) Strongly Disagree

(2) Disagree

(3) Neutral

(4) Agree

(5) Strongly Agree

24. I believe in the importance of sustainable behavior for creating a better world.

- AV3:

(1) Strongly Disagree

(2) Disagree

(3) Neutral

(4) Agree

(5) Strongly Agree

25. I have self-efficacy in engaging in sustainable actions.

- PBC1:

(1) Strongly Disagree

(2) Disagree

(3) Neutral

(4) Agree

(5) Strongly Agree

26. I believe in my personal agency to make a difference through individual behaviors.

- PBC2:

(1) Strongly Disagree

(2) Disagree

(3) Neutral

(4) Agree

(5) Strongly Agree

27. I am confident in my ability to overcome barriers and challenges in practicing sustainability.

- PBC3:

(1) Strongly Disagree

(2) Disagree

(3) Neutral

(4) Agree

(5) Strongly Agree

28. I perceive social expectations regarding sustainable behavior.

- SN1:

(1) Strongly Disagree

(2) Disagree

(3) Neutral

(4) Agree

(5) Strongly Agree

29. Peers, family, and community influence me in promoting or discouraging sustainable actions.

- SN2:

(1) Strongly Disagree

(2) Disagree

(3) Neutral

(4) Agree

(5) Strongly Agree

30. I have role models who exhibit sustainable behaviors and inspire others to follow suit.

- SN3:

(1) Strongly Disagree

(2) Disagree

(3) Neutral

(4) Agree

(5) Strongly Agree

31. I am intrinsically motivated to engage in sustainable behavior based on my personal values and beliefs.

- MI1:

(1) Strongly Disagree

(2) Disagree

(3) Neutral

(4) Agree

(5) Strongly Agree

32. Extrinsic incentives, such as recognition, rewards, or social approval, encourage me to adopt sustainable actions.

- MI2:

(1) Strongly Disagree

(2) Disagree

(3) Neutral

(4) Agree

(5) Strongly Agree

33. I set goals and commit to sustainability-related targets and initiatives.

- MI3:

(1) Strongly Disagree

(2) Disagree

(3) Neutral

(4) Agree

(5) Strongly Agree

34. I have a sense of belonging to a community that values sustainability.

- SIC1:

(1) Strongly Disagree

(2) Disagree

(3) Neutral

(4) Agree

(5) Strongly Agree

35. I identify with environmental and sustainability-related groups or causes.

- SIC2:

(1) Strongly Disagree

(2) Disagree

(3) Neutral

(4) Agree

(5) Strongly Agree

36. I perceive social support for sustainable behavior.

- SIC3:

(1) Strongly Disagree

(2) Disagree

(3) Neutral

(4) Agree

(5) Strongly Agree

37. I have a good understanding and awareness of sustainability issues, including environmental challenges, social responsibility, and economic implications.

- SB1:

(1) Strongly Disagree

(2) Disagree

(3) Neutral

(4) Agree

(5) Strongly Agree

38. I actively engage in behaviors related to waste reduction, recycling, and responsible waste management practices both at school and in my personal life.

- SB2:

(1) Strongly Disagree

(2) Disagree

(3) Neutral

(4) Agree

(5) Strongly Agree

39. I take actions to conserve energy and natural resources, such as turning off lights when not in use, using energy-efficient devices, and minimizing water consumption.

- SB3:

(1) Strongly Disagree

(2) Disagree

(3) Neutral

(4) Agree

(5) Strongly Agree

40. I make choices in transportation modes that have a lower environmental impact, such as walking, cycling, using public transportation, or carpooling.

- SB4:

(1) Strongly Disagree

(2) Disagree

(3) Neutral

(4) Agree

(5) Strongly Agree

41. I consider environmental factors in my purchasing decisions and consumption patterns, choosing environmentally friendly products and reducing single-use items.

- SB5:

(1) Strongly Disagree

(2) Disagree

(3) Neutral

(4) Agree

(5) Strongly Agree

42. I actively participate in sustainability-related activities, such as joining environmental clubs, advocating for sustainable policies, and engaging in community service projects focused on sustainability.

- SB6:

(1) Strongly Disagree

(2) Disagree

(3) Neutral

(4) Agree

(5) Strongly Agree

43. I feel a sense of responsibility and actively contribute to protecting and preserving the natural environment, including activities such as planting trees and promoting biodiversity conservation.

- SB7:

(1) Strongly Disagree

(2) Disagree

(3) Neutral

(4) Agree

(5) Strongly Agree

44. I can effectively collaborate with peers, teachers, and other stakeholders to address sustainability challenges and implement sustainable initiatives within the school community.

- SB8:

(1) Strongly Disagree

(2) Disagree

(3) Neutral

(4) Agree

(5) Strongly Agree
